# Nephroprotective Effect of Heparanase in Experimental Nephrotic Syndrome

**DOI:** 10.1371/journal.pone.0119610

**Published:** 2015-03-18

**Authors:** Suheir Assady, Joel Alter, Elena Axelman, Yaniv Zohar, Edmond Sabo, Michael Litvak, Marielle Kaplan, Neta Ilan, Israel Vlodavsky, Zaid Abassi

**Affiliations:** 1 Department of Nephrology, Rambam Health Care Campus, Haifa, Israel; 2 Department of Pathology, Rambam Health Care Campus, Haifa, Israel; 3 Clinical Laboratories Division, Rambam Health Care Campus, Haifa, Israel; 4 Cancer and Vascular Biology Research Centre, Rappaport Faculty of Medicine, Technion—Israel Institute of Technology, Haifa, Israel; 5 Research Unit, Rambam Health Care Campus, Haifa, Israel; 6 Department of Physiology, Rappaport Faculty of Medicine, Technion—Israel Institute of Technology, Haifa, Israel; UCL Institute of Child Health, UNITED KINGDOM

## Abstract

**Background:**

Heparanase, an endoglycosidase that cleaves heparan sulfate (HS), is involved in various biologic processes. Recently, an association between heparanase and glomerular injury was suggested. The present study examines the involvement of heparanase in the pathogenesis of Adriamycin-induced nephrotic syndrome (ADR-NS) in a mouse model.

**Methods:**

BALB/c wild-type (*wt*) mice and heparanase overexpressing transgenic mice (*hpa*-TG) were tail-vein injected with either Adriamycin (ADR, 10 mg/kg) or vehicle. Albuminuria was investigated at days 0, 7, and 14 thereafter. Mice were sacrificed at day 15, and kidneys were harvested for various analyses: structure and ultrastructure alterations, podocyte proteins expression, and heparanase enzymatic activity.

**Results:**

ADR-injected *wt* mice developed severe albuminuria, while ADR-*hpa*-TG mice showed only a mild elevation in urinary albumin excretion. In parallel, light microscopy of stained cross sections of kidneys from ADR-injected *wt* mice, but not *hpa*-TG mice, showed mild to severe glomerular and tubular damage. Western blot and immunofluorescence analyses revealed significant reduction in nephrin and podocin protein expression in ADR-*wt* mice, but not in ADR-*hpa*-TG mice. These results were substantiated by electron-microscopy findings showing massive foot process effacement in injected ADR-*wt* mice, in contrast to largely preserved integrity of podocyte architecture in ADR-*hpa*-TG mice.

**Conclusions:**

Our results suggest that heparanase may play a nephroprotective role in ADR-NS, most likely independently of HS degradation. Moreover, *hpa*-TG mice comprise an invaluable *in vivo* platform to investigate the interplay between heparanase and glomerular injury.

## Introduction

Glomerular diseases (GDs) are encountered frequently in clinical practice and are the most common cause of end-stage renal disease worldwide [1,2]. It comprises a heterogeneous group of diseases affecting primarily the glomeruli. However, an accompanying injury to kidney vascular and tubulointerstitial compartments is also evident, which may influence therapy, kidney and patient prognosis. Proteinuria, including albuminuria, is one of the most important signs of GDs, indicating a disruption of the normal architecture and/or function of the glomerular filtration barrier (GFB). In addition, proteinuria signifies a greater likelihood of chronic kidney disease (CKD) to progress to more advanced stages, and is also a risk factor for cardiovascular disease, non-dipping hypertension, and all-cause mortality [3,4].

Mammalian heparanase is an endo-β(1,4)-D-glucuronidase that degrades heparan sulfate (HS) side chains of heparan sulfate proteoglycans (HSPGs). It is associated among others with extracellular matrix (ECM) turnover, angiogenesis, thrombosis, inflammation, autoimmunity, and cancer metastasis [5–9]. Emerging evidence suggests the involvement of heparanase in diabetic and non-diabetic proteinuric kidney diseases [10,11]. For instance, heparanase expression was shown to be upregulated in a number of animal models of renal disease: passive Heymann nephritis [12], puromycin aminonucleoside nephrosis (PAN) [13], Adriamycin nephropathy (ADR-N) [14,15], anti-glomerular basement membrane (GBM) nephritis [16], and diabetic nephropathy [17,18]; and in renal epithelial and endothelial cells cultured in ambient high glucose concentration [18]. As expected, heparanase upregulation was associated with a reduced HS size in the GBM. Likewise, increased heparanase activity was detected in urine samples from diabetic patients with microalbuminuria [19–21], non-diabetic nephrotic syndrome, CKD and kidney transplanted patients [19]. In ADR-N, it was postulated that an interplay between heparanase/HS and reactive oxygen species (ROS)/Angiotensin II (AII)/aldosterone axis is involved in modulation of the GBM permeability and associated proteinuria [14,15]. Such effects were partially reversed using ROS scavenger and AII receptor blocker. Interestingly, neutralization of heparanase activity, using either a sulfated oligosaccharide inhibitor (PI-88) or anti-heparanase antibodies, resulted in reduced proteinuria [22]. Similar findings were reported by Gil et al [23] and Goldberg et al [24] who demonstrated that heparanase null mice fail to develop albuminuria and renal damage in response to streptozotocin-induced diabetes mellitus.

Zcharia and colleagues established a transgenic mouse strain (*hpa*-TG) overexpressing human heparanase in all tissues [7]. Immunostaining of kidneys confirmed the overexpression of heparanase, accompanied by a marked decrease in HS and two fold increase in proteinuria vs. controls. These mice were fertile and exhibited a normal life span. Accordingly, we assumed that *hpa*-TG mice are an ideal experimental platform to elucidate the involvement of heparanase in the pathogenesis of GFB injury and proteinuria. The glomerular injury was induced by injection of Adriamycin, a well-characterized and established model for investigating human focal segmental glomerulosclerosis [25,26]. To our surprise, mice constitutively expressing the human heparanase gene were resistant to Adriamycin nephrotoxic effects.

## Materials and Methods

### Experimental animals

Wild type (*wt*) male BALB/c mice, 10–12-weeks old, were purchased from Harlan Laboratories (Jerusalem, Israel). Male, homozygous *hpa*-TG mice, in which the human heparanase gene is driven by a constitutive β-actin promoter in a BALB/c genetic background, were bred at our animal facility at the Rappaport Faculty of Medicine, Technion, Israel Institute of Technology, as have been previously described [7]. *hpa*-TG mice were crossed for 10 generations with BALAB/c mice to produce pure genetic background [27]. All mice (n = 80) were maintained under conventional pathogen-free conditions, in a temperature-controlled room, and fed with standard mouse chow and tap water *ad libitum*. All studies were performed according to the protocol approved by the Technion Animal Inspection Committee.

To induce nephrotic syndrome, mice were held in a restrainer and received a single dose of Adriamycin (10 mg/kg), via a tail vein injection. Mice administered with 0.9% sodium chloride solution served as controls. For urine collection, mice were housed in metabolic cages for 24 hours, at days 0, 7, and 14 after Adriamycin injection. Animals from the various experimental groups were anesthetized (Pentobarbitone sodium, 60 mg/kg, i.p.) at day 15, blood samples were drawn via cardiac puncture, and kidneys were harvested. Kidneys were immediately either frozen in liquid nitrogen and stored in -80°C for protein analysis, or stored in fixatives (see below). It should be emphasized that the animals remained fully anesthetized for the entire duration of this procedure, up to and including death, as a result of either hemorrhage or euthanasia, and all efforts were made to minimize suffering.

### Sera and urinary samples

Serum biochemistry parameters were determined using commercial kits on the diagnostic analyzer Dimension RXL (Siemens, Germany). Dedicated reagents kits were used for the measurement of total cholesterol (DF27), triglycerides (DF69A), and Albumin (DF13). Urine samples were stored at −20°C until assayed for urinary albumin using an ELISA assay (Cayman Chemicals, Ann Arbor, MI), and urinary creatinine using a colorimetric kit assay based on the Jaffe reaction (Cayman Chemicals). The albumin to creatinine ratio was determined to deduce the extent of albuminuria.

### Renal heparanase enzymatic activity

Heparanase activity was evaluated as described before [6,7,28,29]. Briefly, equal amounts of protein derived from renal cortex lysates were incubated for 18 hours at 37°C, pH 6.2–6.6, with ^35^S-labeled ECM. The incubation medium was centrifuged and the supernatant was analyzed by gel filtration on a Sepharose CL-6B column (0.9x30 cm). Fractions (0.2 ml) were eluted with PBS and their radioactivity was measured. Intact HSPGs are eluted just after the void volume (fractions 1–10), whereas HS degradation fragments are eluted toward the Vt of the column (fractions 15–35; 0.5<Kav<0.8).

### Renal histopathology

Kidney tissues were fixed in 10% neutral-buffered formalin (NBF), progressively dehydrated in graduated alcohol concentrations (70–100%) and embedded in paraffin. For general histomorphology, 5 μm-sections were stained with hematoxylin and eosin (H&E), periodic acid-Schiff (PAS), and Masson's trichrome reagents.

### Immunofluorescence

Whole kidneys were rapidly frozen in liquid nitrogen, and 5 μm-thick cryostat sections were placed on silane-coated slides and dried at room temperature. The samples were blocked with 10% normal goat serum (NGS) in phosphate-buffered saline (PBS) at room temperature for 1 h. Then, they were incubated, overnight at 4°C, with either polyclonal rabbit anti nephrin (1:150, kindly provided by Dr. D. Salant, Boston) or anti podocin (1:100, Sigma Aldrich). Slides were washed and incubated with secondary antibodies: Dylight 488-conjugated goat anti-rabbit IgG (1:500, Jackson ImmunoResearch Laboratories). Nuclei were counterstained with 4’,6-diamidino-2-phenylindole (DAPI). Immunofluorescence images were viewed with a Zeiss Axioscope 2 fluorescent upright microscope, and digital images were captured with a high sensitive black and white, charged-coupled device (CCD) camera (Olympus DP70), controlled by Image-Pro software (Media Cybernetes, Rockville, MD), with fixed settings. Confocal microscopy was performed using the Zeiss LSM 510 Meta scanning system.

Analysis and pseudocolour rendering were carried out using Image-Pro Plus ver. 7 software. A threshold for the positively fluorescent staining per image was established. The optical density of the staining was represented by the average of the gray level pixels per glomerulus with values ranging from 0 (unstained pixels) to 255 (strongest stained pixels).

### Ultrastructure assessment and morphometry

Kidney cortices were fixed in 3.5% glutaraldehyde and rinsed in 0.1 M sodium cacodylate buffer, pH 7.4. Tissue blocks (1 mm^3^) were post-fixed with 2% OsO_4_ in 0.2 M cacodylate buffer for 1 h, rinsed again in cacodylate buffer to remove excess osmium, immersed in saturated aqueous uranyl acetate, dehydrated in graded alcohol solutions, immersed in propylene oxide, and embedded in Epon 812. Ultrathin sections (80 nm) were mounted on 300-mesh, thin-bar copper grid, counterstained with saturated uranyl acetate and lead citrate. Sections were examined with a transmission electron microscope (Jeol 1011 JEM), at 80 KV.

The quantification of podocyte effacement was performed as previously described [30]. In brief, the length of the peripheral GBM was measured at X 5000 and X 15000 magnification and the number of slit pores overlying the GBM length was counted. The arithmetic mean of foot process width (W_FP_) per glomerulus was calculated as the total (Σ) GBM length measured in one glomerulus divided by the total number (Σ) of slits counted, then multiplied by π/4, a correction factor for the random orientation by which the foot processes were sectioned:

$$W_{FP}=\left(\Sigma GBM\;length/\Sigma Slits\right)x\pi /4$$

To visualize anionic sites along the GBM, Polyethyleneimine (PEI, 1.8 kDa) labeling was conducted as previously described [31].

### Western blotting

Renal cortex tissue samples, from three experiments, were homogenized on ice and centrifuged at 4°C for 5 min at 3000 RPM. In additional two experiments, glomeruli were isolated by using commercial Dynabeads M-450 Tosylactivated beads (Invitrogen, Carlsbad, CA), as previously described [32]. The homogenized tissue or isolated glomeruli were lysed in RIPA buffer (150 mM NaCl, 1% NP40, 50 mM Tris pH 8.0, 0.5% sodium deoxycholate and 0.1% SDS) in rotation at 4°C for 30 min, and then centrifuged at 4°C for 10 min at 12000 RPM. The cleared supernatant was collected and protein concentration was determined by the Bio-Rad protein assay. Equal amounts of extracted proteins (20–60 μg) were resolved by electrophoresis on a 7.5–10% SDS—polyacrylamide gel, and were transferred to nitrocellulose membranes. The membranes were incubated in blocking buffer, TBS-T (Tris-buffered saline and 0.1% Tween 20) containing 5% (w/v) nonfat dry milk, and probed with the appropriate primary antibodies: polyclonal rabbit anti nephrin (1:3000, kindly provided by Dr. D. Salant, Boston), anti podocin (1:1000, Sigma Aldrich), anti heparanase 733 (1:1000) [33], or anti P97 (1:2000, a gift from Dr. A. Stanhill, Haifa). After washing with TBS-T, the immunoreactive proteins were visualized with horseradish-conjugated IgG (Jackson ImmunoResearch Laboratories) diluted 1:10,000 and an enhanced chemiluminescence system (WesternBright, Advansta).

### Statistical analysis

Data are presented as mean of repeated measurements ± standard error (S.E.M). Comparison between two parametric groups was done using the unpaired Student T test after testing for the equality of variances. More than two paired groups were tested using the one-way analysis of variance (ANOVA) test for repeated measurements, followed by the Bonferroni post-hoc test for multiple comparisons. Association between categorical groups after finding best cutoff points were tested using the Chi square or the Fisher's exact test as needed. Two tailed P values of 0.05 or less were considered to be statistically significant.

## Results

### *hpa-*TG mice are resistant to Adriamycin-induced albuminuria

**Fig 1:**
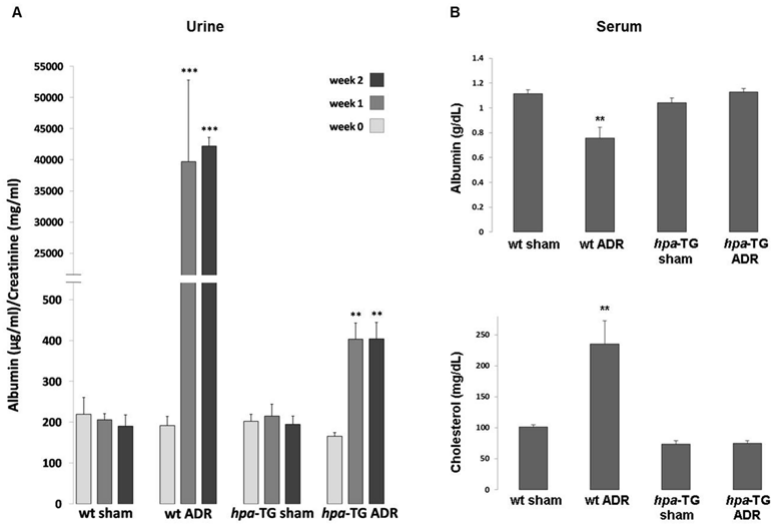
*hpa*-TG mice are protected from Adriamycin-induced albuminuria and nephrotic syndrome. **(A)** Male wild type BALB/c mice (*wt*) and *hpa*-TG mice were injected with Adriamycin (ADR) or kept as control (sham). Prior to injection (week 0) or one and two weeks post injection, urine was collected for 24h and used to determine albumin/creatinine ratio concentrations as described (n = 7 per each experimental group). **, P<0.001 vs. *hpa*-TG sham; ***, P<0.0001 vs. *wt* sham. **(B)** Blood samples were drawn from cardiac puncture at the day of sacrifice. Sera were analyzed for albumin and cholesterol (n = 13, 9, 12, and 11 for *wt* sham, *wt* ADR, *hpa*-TG sham, and *hpa*-TG ADR mice, respectively). **, P<0.001 vs. *wt* sham.

To investigate the involvement of heparanase in ADR-N experimental model, *hpa*-TG mice and *wt* BALB/c control mice were injected with a single dose of Adriamycin (10 mg/kg). To assess albuminuria, a hallmark of GFB injury, urine samples were collected for 24 hours, prior to Adriamycin injection (baseline values), and one and two weeks after Adriamycin administration. As depicted in Fig. 1A, urinary albumin/creatinine ratio (ACR) increased after induction of injury, in both *hpa*-TG mice and *wt* mice. However, the increase was remarkably greater in the latter compared with their non-injected counterparts (ACR- 0.19 ± 0.02 vs. 0.40 ± 0.041 mg/mg, p = 0.004, *hpa*-TG sham vs. *hpa*-TG ADR, respectively; and 0.19 ± 0.03 vs. 42.24 ± 1.39 mg/mg, p <0.0001, *wt* sham vs. *wt* ADR, respectively, at 14 days after Adriamycin injection). The severity of albuminuria in these groups corresponds with serum albumin levels, which were significantly lower in the *wt* ADR as compared with the *hpa*-TG ADR group, at day 15 (Fig. 1B). Similar to clinical setting, nephrotic *wt* mice also displayed severe hypercholesterolemia, which was not evident in the *hpa*-TG ADR group (Fig. 1B).

Of note, in a qualitative assay, heparanase enzymatic activity was enhanced following Adriamycin-induced injury in *wt* mice (S1 Fig.). When incubated with sulfate-labeled ECM, extracts from cortices of ADR injected *wt* mice resulted in release of high amounts of HS degradation fragments (Adria, peak at fraction 24) as compared with untreated control mice (sham). Nevertheless, overexpression of heparanase in *hpa-*TG mice was associated with mild proteinuric response to Adriamycin administration, suggesting heparanase independent mechanisms of injury in wild type mice.

### Adriamycin injection results in reduced expression of podocyte-specific markers in wild type mice but not in *hpa*-TG mice

**Fig 2:**
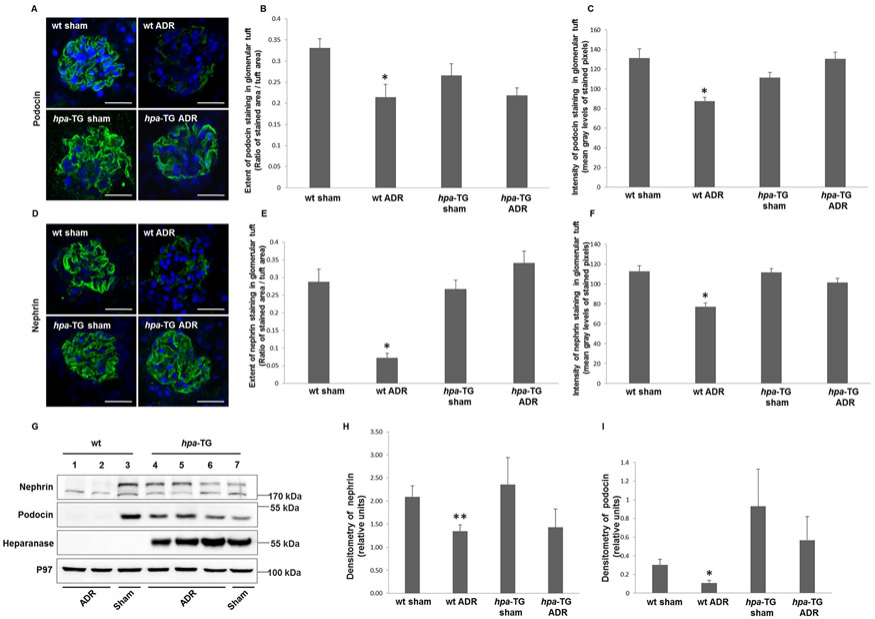
Expression of key slit diaphragm proteins is reduced in wild type but not in *hpa*-TG Adriamycin-injected mice. Podocyte markers expression is reduced in wild type but not in *hpa*-TG Adriamycin-injected mice. *wt* or *hpa*-TG mice were injected with Adriamycin (ADR) or served as control (sham). Two weeks post injection, the animals were sacrificed. **(A, D**) Representative immunofluorescence staining performed on cryostat sections from kidneys of the indicated experimental groups, using anti-podocin or anti-nephrin primary antibodies, and Dylight 488-conjugated anti-rabbit IgG. Nuclei were stained with DAPI (Confocal microscope, scale bar = 25 μm). The extent of podocin **(B)** and nephrin **(E)** staining and its intensity **(C, F)** in glomerular tuft were quantified. For podocin we analyzed n = 30, 23, 15 and 24 glomeruli, for *wt* sham, *wt* ADR, *hpa*-TG sham, and *hpa*-TG ADR mice, respectively), whereas for nephrin n = 22, 23, 17 and 27 glomeruli, for *wt* sham, *wt* ADR, *hpa*-TG sham, and *hpa*-TG ADR mice, respectively. Glomeruli were evaluated at least from 3–4 mice per each experimental group. *, P<0.01 vs. *wt* sham. **(G**) Protein was extracted from kidney cortex or isolated glomeruli and analyzed by Western blotting using the indicated antibodies. P97 was used as loading control. A representative immunoblot is depicted in panel G (n = 7, 11, 7 and 11 for *wt* sham, *wt* ADR, *hpa*-TG sham, and *hpa*-TG ADR mice, respectively from three independent experiments) **(H, I)** Densitometry of immunoblotting of isolated glomeruli lysates (n = 8, 7, 3 and 6 for *wt* sham, *wt* ADR, *hpa*-TG sham, and *hpa*-TG ADR mice, respectively). *, P = 0.03 vs. *wt* sham **, P = 0.02 vs. *wt* sham.

Renal damage in rodents exposed to Adriamycin has previously been linked to podocyte injury [26]. To assess whether heparanase might possess a direct protective role in these cells, expression of podocyte markers was determined, applying Western blot and immunofluorescence analyses. Expression of podocin and nephrin, key podocyte-specific markers, was dramatically reduced in renal cortex and glomeruli of *wt* mice subjected to Adriamycin administration compared with their controls. In contrast, injected *hpa*-TG mice exhibited mild non-significant alterations in podocin or nephrin expression as compared with non-injected *hpa*-TG mice (Fig. 2 G-I).

To further validate these findings at the histological level, kidney cross sections were immunostained for podocin and nephrin (Fig. 2A, D). In agreement with the above mentioned results, immunoreactive levels of both markers were dramatically reduced in glomerular tufts derived from Adriamycin-injected *wt* mice but not in *hpa*-TG mice administered with Adriamycin (Fig. 2 A-F).

### Adriamycin injection leads to podocyte injury in wild type mice but not in *hpa*-TG mice

In light of the different nephrin and podocin abundance between *wt* and *hpa-*TG mice following Adriamycin injection, two approaches were taken to determine the structural and ultrastructural glomerular damage utilizing light and electron microscopy, respectively.

**Fig 3:**
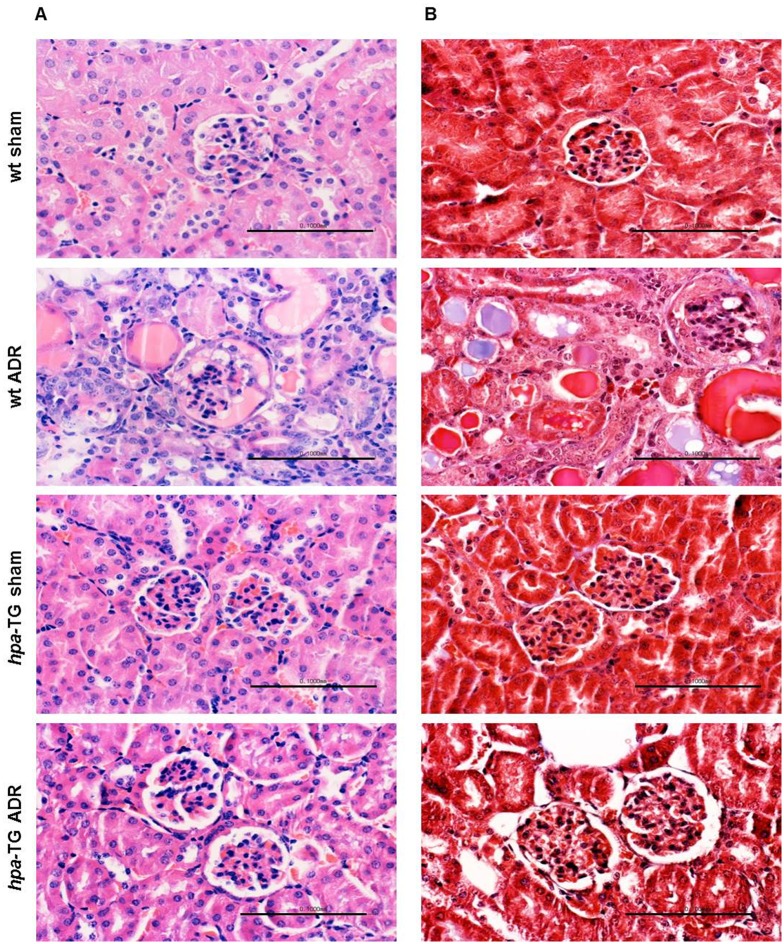
Adriamycin causes renal damage in wild type mice but not in *hpa*-TG mice. *wt* or *hpa*-TG mice were injected with Adriamycin (ADR) or served as control (sham). Two weeks post injection, the animals were sacrificed. Representative images of H&E **(A)** and Masson’s trichrome **(B)** staining of paraffin-embedded kidney sections from various experimental groups (scale bar = 100 μm). n = 6 mice per each experimental group, from three independent experiments.

When blindly observed by light microscopy, H&E and PAS stained cross sections of paraffin-embedded *hpa*-TG kidneys, both Adriamycin-injected and sham animals, could not be distinguished from those obtained from healthy BALB/c mice. The glomerular capillary loops were open, and tubulointerstitial structure appeared normal. Conversely, cross sections from Adriamycin-injected wild type mice showed variable degrees of glomerular and tubular damage (mild to severe), as evident by the presence of a proteinatious material inside the urinary space and tubular lumen, as well as tubular atrophy and dilation (Fig. 3A). However, two weeks following Adriamycin-induced injury, no signs of interstitial fibrosis were detected by Masson’s trichrome staining (Fig. 3B).

**Fig 4:**
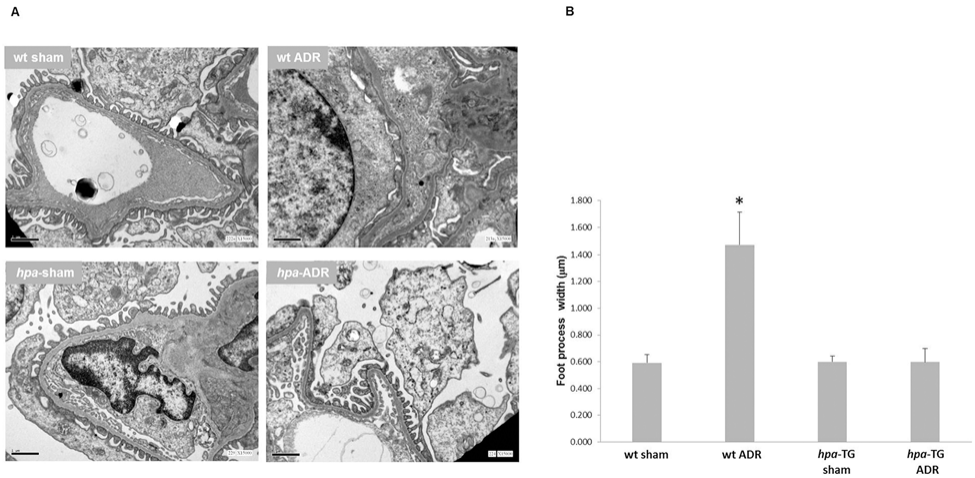
Heparanase protects podocytes from Adriamycin induced injury. *wt* or *hpa*-TG mice were injected with Adriamycin (ADR) or served as control (sham). Two weeks post injection, the animals were sacrificed. **(A)** Representative images of transmission electron microscopy of ultrathin sections of kidney tissue. Magnification X15 000; scale bar = 1μm. **(B)** Quantification of foot process width (n = 12 glomeruli per each wt group, 13 for *hpa*-TG, and 11 for *hpa*-TG ADR group, obtained from 6, 3, 4, and 5 animals, respectively). *, P<0.01 vs. all other groups.

This approach, though a hallmark characteristic of glomerular injury, does not necessarily indicate podocytes’ fate. Hence, electron microscopy was applied. In line with the observed massive proteinuria, Adriamycin-injected wild type mice demonstrated flattening and effacement of foot processes (Fig. 4). While, analysis of ultrathin kidney sections of injected *hpa*-TG mice revealed normal podocyte architecture with numerous, intact foot processes and slit diaphragm (Fig. 4). Mean foot process width was significantly increased (1470 ± 244 nm) in ADR-injected *wt* mice vs. *wt* controls, ADR-injected and control *hpa*-TG mice (593 ± 60 nm, 599 ± 100 nm, and 600 ± 41 nm, respectively, Fig. 4B). Furthermore, in pilot experiments where PEI staining was used, we demonstrated that *hpa*-TG mice displayed a decrease in the GBM anionic charge as compared with *wt* controls. In addition, disruption of these anionic sites was observed in both ADR-injected *wt* and *hpa*-TG mice (S2 Fig.). Such a reduction in negative charge sites did not correlate with the degree of albuminuria presented in Fig. 1A, suggesting a distinctive role of heparanase not related to charge permselectivity or HSPGs.

## Discussion

The data reported in the present study provide important information on the involvement of heparanase in the pathogenesis of glomerular injury, and extend our previous understanding regarding mechanisms underlying the development of proteinuria in nephrotic syndrome. Our findings clearly demonstrate that ADR-*hpa*-TG mice exhibited only mild albuminuria as compared with severe urinary protein excretion obtained in ADR-injected *wt* mice. The development of proteinuria in the latter was associated with glomerular and tubular damage as compared with normal kidney structure in ADR-*hpa*-TG mice. In line with these findings, Western blot of both cortical homogenate and isolated glomeruli lysate and immunofluorescence analyses revealed profound reduction in nephrin and podocin expression in ADR-*wt* mice, but not in ADR-*hpa*-TG mice. Furthermore, electron-microscopy revealed significant podocyte loss, and foot process effacement in injected ADR-*wt* mice, as compared with largely preserved integrity of podocyte network in ADR-*hpa*-TG mice. Collectively, these results suggest that heparanase exerts a nephroprotective effect in ADR-NS.

Nonetheless, as indicated in Fig. 1, the degree of basal albuminuria in our *hpa*-TG mice was comparable to *wt* controls. Previous studies on *hpa*-TG mice that were generated in C57BL genetic background documented normal kidney function associated with variable degree of proteinuria, ranging from high normal to mildly elevated magnitude [7,34]. Of note, in our study we only used BALB/c mice in order to eliminate possible genetic confounding factors, which were shown to influence the sensitivity to Adriamycin [26]. Specifically, C57BL mice were resistant to the deleterious renal impact of Adriamycin, as well as other proteinuric experimental models.

Proteinuria, an important sign of many primary and secondary GDs, usually indicates an injury to one or more of the GFB layers [35]. Although recent years have known major breakthroughs related to the pathogenesis of proteinuria [36–38], the molecular mechanisms underlying this phenomenon remain poorly elucidated. This issue is of special interest since a comprehensive understanding of the GFB and proteinuria could potentially lead to introducing novel, mechanism-specific therapies. While the contribution of nephrin and podocin, two of the slit diaphragm structural proteins, for maintaining functional glomerular filtration barrier is well established, the involvement of HS and heparanase is still evolving and even debatable [9,10,31,39,40]. Therefore, heparanase genetically manipulated (*hpa*-TG) mice are valuable tool to address the involvement of heparanase in proteinuric diseases.

HSPGs are associated with, and ubiquitously present on the cell surface and extracellular matrix of a wide range of mammalian cells and tissues, including glomeruli [9,41,42]. The interactions of HSPGs with other ECM macromolecules, and with different attachment sites on the cell membrane indicate that HS play a key role in structural integrity, self-assembly, insolubility, and permselective properties of basement membranes and ECM, and in cell adhesion and locomotion [43]. Heparanase is the only mammalian enzyme that degrades HS. Hence, the previously reported upregulation of heparanase in diverse experimental and clinical kidney injury may implicate this enzyme in the pathogenesis of GDs. In line with previous reports [14,15], we demonstrate an increase of heparanase activity following induction of nephrotic syndrome using Adriamycin in *wt* control mice. In contrast to *wt* mice, *hpa*-TG animals were resistant to the adverse renal and metabolic effects of Adriamycin, as was evident by low magnitude proteinuria and lack of histological alterations in the renal tissue. Since proteinuria is a major hallmark of various GDs, our findings suggest, for the first time, a nephro-protective role of heparanase during nephropathy progression in proteinuric diseases of various etiologies. Our results are concordant with the publication of van den Hoven et al, [11], who demonstrated preservation of foot processes of podocytes in *hpa*-TG mice. However, our findings are at odds with the original publication by Zcharia et al [7], who demonstrated that overexpression of heparanase resulted in increased levels of urinary protein and serum creatinine, suggesting an adverse effect on kidney function as reflected also by electron microscopy podocyte foot process effacement. However, the mice that were used by these authors are with mixed genetic background and no quantification of foot process width was performed. The angle by which the foot process was sectioned might also affect the interpretation.

The cellular and molecular mechanisms responsible for the beneficial effects of heparanase are not known, and are subject of future studies. Our results clearly show that *hpa*-TG mice subjected to Adriamycin administration are characterized by well-maintained GFB, both at the functional and structural levels, especially the key slit diaphragm proteins, nephrin and podocin, as compared to ADR-*wt* controls. The lack of nephrin/podocin disruption in *hpa*-TG ADR may contribute to intact size permselectivity of the GFB. Additional possibility is related to charge permselectivity and its role in the development of proteinuria. Noteworthy, the primary role of HS in the charge selectivity of the GBM has been recently debated. In this regard, *in vivo* degradation of the GBM-HS did not result in proteinuria, and moreover, disruption of GBM charge through podocyte-specific knockout of *Agrn* or *Ext1* genes did not influence glomerular permselectivity [31,39,40]. Similarly, a prospective clinical trial in microalbuminuric diabetic patients failed to demonstrate any beneficial effect of Sulodexide, a heterogeneous group of sulfated glycosaminoglycans [44]. Our results are in agreement with these observations, because the reduced anionic sites along the GBM of *hpa*-TG mice, as we and others [34] have demonstrated, did not translate into heavier proteinuria neither in health nor in disease. Hence, the question is whether the previously observed alterations in HS/heparanase expression in proteinuric diseases could possibly be a consequence rather than a cause of proteinuria.

Interpretation of the observed effects of heparanase on kidneys, especially in *hpa*-TG mice, could be attributed to either systemic or local effects of heparanase, because the transgene is driven by a constitutive β-actin promoter, and expressed in all mouse tissues. A podocyte-targeted heparanase overexpressing mouse would be a preferable model, however such a model has not yet been generated. In the present study, we mainly focused on glomerular damage. Yet, preserved proximal tubular function may also contribute to minimal proteinuria observed in the transgenic mice. On the other hand, ADR-nephropathy is a robust model accompanied by massive proteinuria, indicating a major GFB disruption. This assumption is supported by the fact that tubular dysfunction usually causes subnephrotic proteinuria. Moreover, the electron microscopy findings strengthen our assumption that the glomerulus is the major site of injury by ADR or alternatively the putative site for protection by heparanase. The effects of heparanase on proximal tubules and other nephron segments worth further investigation, especially in light of recent studies, which emphasize the role of the proximal tubule in ultrafiltrate albumin reabsorption and reclamation (reviewed in [45]).

Interestingly, in recent years there is increasing evidence that enzymatically inactive heparanase promotes vascular endothelial growth factor (VEGF), hepatocyte growth factor, and tissue factor expression, as well as Akt, Src and EGF-receptor phosphorylation [46], emphasizing the notion that non-enzymatic activities of heparanase play a significant role in heparanase biological actions. Therefore, one can speculate that heparanase may indirectly affect glomerular health through upregulation of VEGF-A, which in turn maintains glomerular endothelial cell (GEnC) fenestrations and glycocalyx [47,48], and thereby counteracting the well-known toxic effects of Adriamycin on GEnCs [49]. In our study, we did not, however, examine the GEnC surface layer because it requires special fixation and staining protocols for transmission electron microscopy [50], which were not applied herein.

In conclusion, the present study provides new insights into the involvement of heparanase in the pathogenesis of proteinuria. Specifically, our results suggest that heparanase may have a nephroprotective role in ADR-NS, most likely *via* a mechanism independent of HS degradation. Moreover, *hpa*-TG mice comprise an invaluable *in vivo* platform to investigate the interplay between heparanase and glomerular injury, which may open new options for future therapeutic interventions.

## Supporting Information

S1 Fig Heparanase enzymatic activity is elevated in wild type mice exposed to Adriamycin. A representative heparanase enzymatic activity assay that was determined two weeks post Adriamycin injection on cortex from control *wt* BALB/c mice (sham) vs. injected mice (Adria.).
(TIF)

S2 Fig Polyethyleneimine (PEI) labeling of anionic sites along the glomerular basement membrane (GBM). To visualize the GBM anionic sites (arrows) of Adriamycin (ADR) injected and uninjected (sham) wild type (*wt*) and transgenic (*hpa*-TG) mice, PEI (1.8 kDa) labeling was conducted as previously described [27]. Transmission electron microscopy, original magnification: X30 000 (n = two animals per each experimental group); scale bar = 0.5 μm.
(TIF)
