# Supplementary figures and images for: Nephroprotective Effect of Heparanase in Experimental Nephrotic Syndrome
Source: PLoS One. 2015 Mar 18;10(3):e0119610. doi: 10.1371/journal.pone.0119610 (PMC4364762; doi:10.1371/journal.pone.0119610)

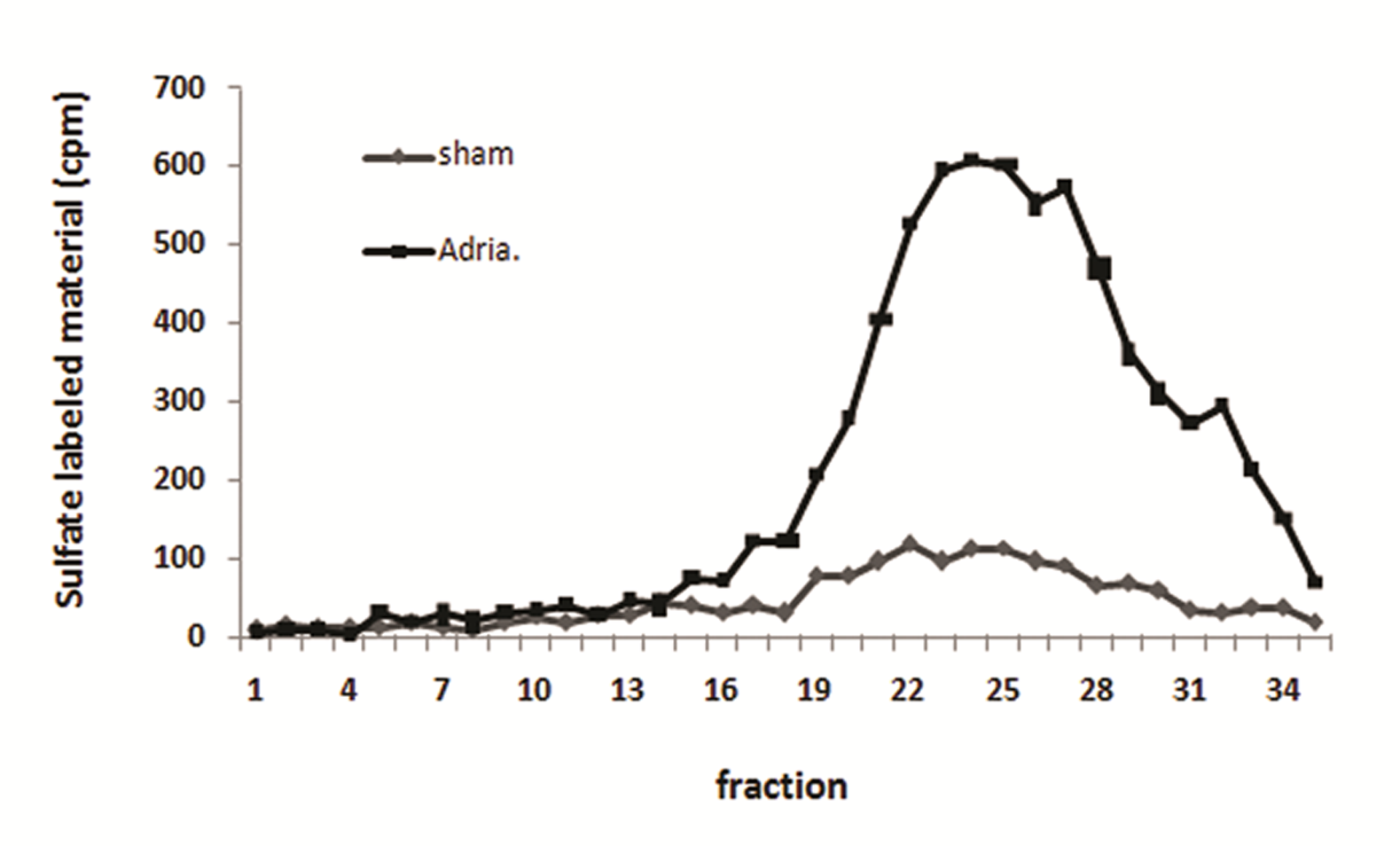

Supplement: S1 Fig — A representative heparanase enzymatic activity assay that was determined two weeks post Adriamycin injection on cortex from control wt BALB/c mice (sham) vs. injected mice (Adria.). (TIF) [file pone.0119610.s001.tif]

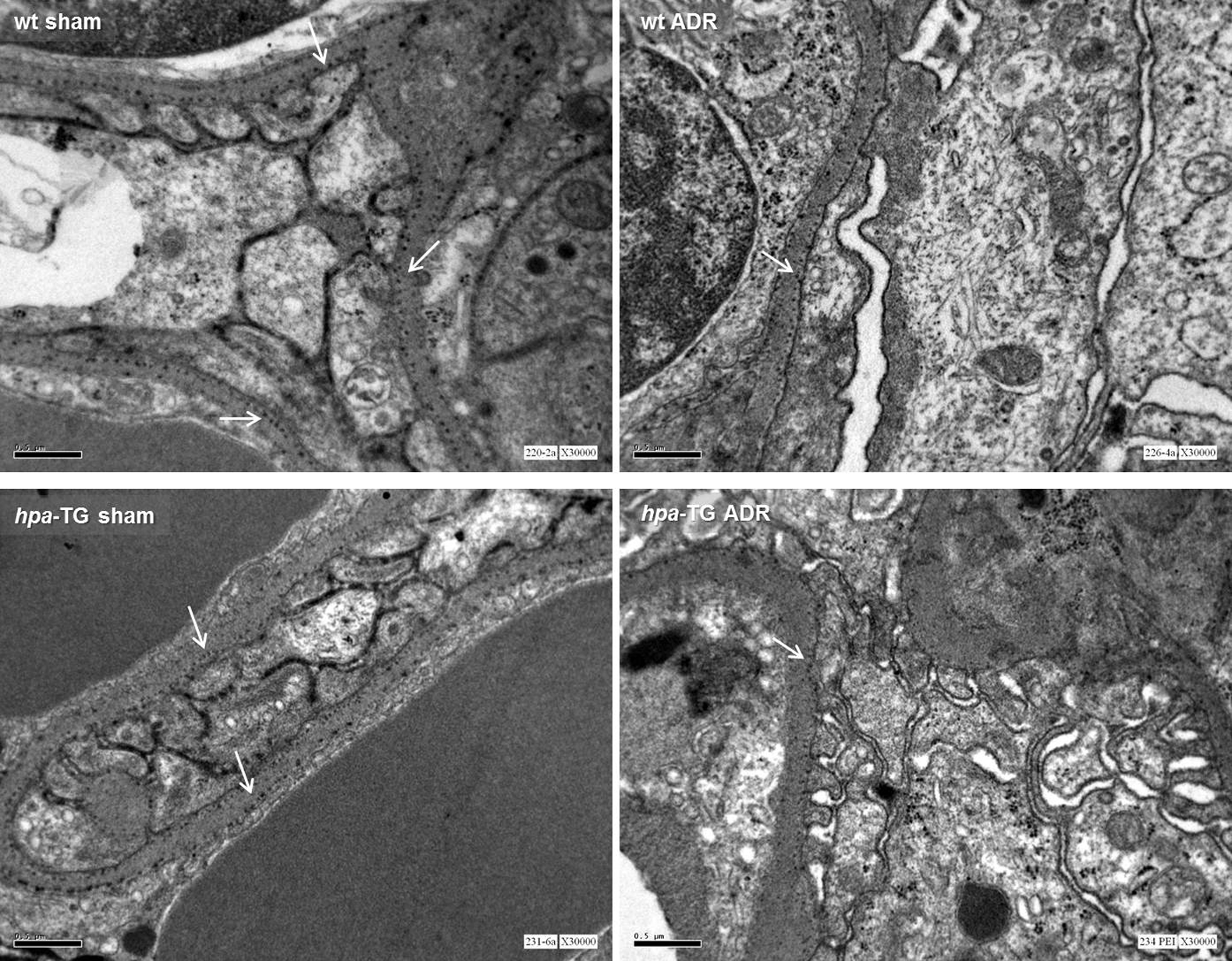

Supplement: S2 Fig — To visualize the GBM anionic sites (arrows) of Adriamycin (ADR) injected and uninjected (sham) wild type (wt) and transgenic (hpa-TG) mice, PEI (1.8 kDa) labeling was conducted as previously described [27]. Transmission electron microscopy, original magnification: X30 000 (n = two animals per each experimental group); scale bar = 0.5 μm. (TIF) [file pone.0119610.s002.tif]
